# Adaptive laboratory evolution – principles and applications for biotechnology

**DOI:** 10.1186/1475-2859-12-64

**Published:** 2013-07-01

**Authors:** Martin Dragosits, Diethard Mattanovich

**Affiliations:** 1 Department of Chemistry, University of Natural Resources and Life Sciences, Muthgasse 11, A-1190 Vienna, Austria; 2 Department of Biotechnology, University of Natural Resources and Life Sciences, Vienna, Austria

**Keywords:** Laboratory evolution, Cell factory engineering, Microbial growth, Microbial stress, Biotechnology

## Abstract

Adaptive laboratory evolution is a frequent method in biological studies to gain insights into the basic mechanisms of molecular evolution and adaptive changes that accumulate in microbial populations during long term selection under specified growth conditions. Although regularly performed for more than 25 years, the advent of transcript and cheap next-generation sequencing technologies has resulted in many recent studies, which successfully applied this technique in order to engineer microbial cells for biotechnological applications. Adaptive laboratory evolution has some major benefits as compared with classical genetic engineering but also some inherent limitations. However, recent studies show how some of the limitations may be overcome in order to successfully incorporate adaptive laboratory evolution in microbial cell factory design. Over the last two decades important insights into nutrient and stress metabolism of relevant model species were acquired, whereas some other aspects such as niche-specific differences of non-conventional cell factories are not completely understood. Altogether the current status and its future perspectives highlight the importance and potential of adaptive laboratory evolution as approach in biotechnological engineering.

## 

Microbial cells are of special importance for biotechnological applications and bacteria and yeasts are used for many biotechnological tasks, ranging from biofuel production [1-3], commodity chemical synthesis [4,5], through to the production of industrial and biopharmaceutical proteins [6-8]. Moreover, biological catalysis offers several advantages over chemical synthesis, such as stereo-selective production of chemical compounds [9]. Synthetic biology is a new emerging field [10,11] and systems metabolic engineering has proven very successful for the design and implementation of biotechnological production processes [12-14]. Recently, classical genetic engineering was successfully complemented with artificial laboratory selection of microbial cells in order to generate potentially robust and optimized microbial production systems [15,16].

Here we summarize the basics and implications of adaptive laboratory evolution for biological engineering. We present important studies on laboratory evolution and evolutionary engineering with the aim of improving microbial growth on relevant substrates and stress resistance. Furthermore, we discuss the existence of cross-stress dependencies in bacteria and yeasts and the implications of evolutionary engineering for non-conventional host organisms. It is clear that the vast amount of available data on this topic exceeds the scope of this recapitulation. Towards this end we want to point out that some other recent reviews discussed the importance of adaptive laboratory evolution (ALE) in applied microbiology and may cover other relevant topics [15-19]. Furthermore, additional reviews cover more general aspects of ALE [20-23].

## Adaptive laboratory evolution

Adaptive laboratory evolution as a scientific approach is very important towards the analysis of evolutionary phenomena in a controlled laboratory setting. The principles on which laboratory evolution experiments are based, date back to scientists such as Antonie van Leeuwenhoek, Louis Pasteur, Robert Koch and most notably Charles Darwin, with their discoveries of microorganisms, the general acceptance of the germ theory and the importance of natural and artificial selection for biological evolution and breeding. Hence, adaptive laboratory evolution was already performed about a hundred years ago by William Dallinger [22] and during the middle of the last century [24,25]; however, particularly in the last 25 years, there has been an ever increasing number of such experiments with *Escherichia coli* and *Saccharomyces cerevisiae* being the most prominent organisms under investigation [26-28].

During microbial ALE, a microorganism is cultivated under clearly defined conditions for prolonged periods of time, in the range of weeks to years, which allows the selection of improved phenotypes. Microbial cells offer important advantages for ALE studies: (a) most microbial cells have simple nutrient requirements, (b) they can be easily cultivated in the laboratory and (c) microbial cells generally grow very fast and can be cultivated for several hundred generations within several weeks or months (with typical specific growth rates of microbial cells in the range of μ = 0.05 to 1.0 h^-1^). In contrast to comparative genomics [29], ALE allows phenotypic changes to be clearly associated with a certain growth environment that leads to the selection of traits. Moreover, due to rather new technologies, including transcriptional profiling [30] and massive next-generation DNA sequencing (NGS) [31,32], phenotype-genotype correlations can be easily obtained by whole genome re-sequencing (WGS). ALE led to important insights and experimental proof for evolutionary biology. On the forefront of laboratory evolution experiments, the long term study of Professor Lenski and his research group at the Michigan State University has to be mentioned. This single parallel *E*. *coli* adaptation experiment is already exceeding 50000 generations [33-35]. Together with other similar experiments, the scientific community was provided with insights into the genetic basis of increased fitness [36], implications of historical contingency in laboratory evolution [37], second order effects during evolution [38], the interrelation of population size, robustness and evolvability [39-41], clonal interference [42] and evolutionary bet hedging [43].

### Laboratory selection methods

**Figure 1:**
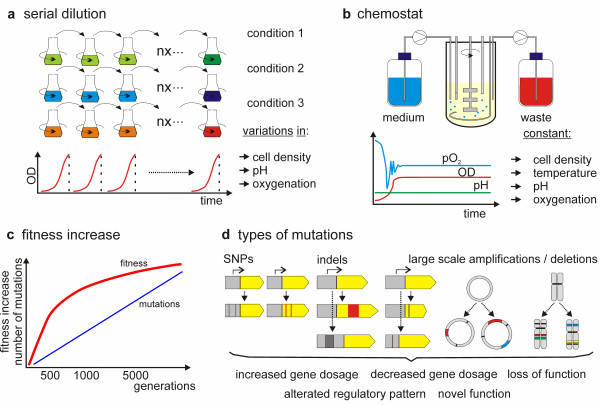
**Adaptive laboratory evolution (ALE).** ALE can be performed in the laboratory by (**a**) sequential serial passages in shake flasks, where nutrients will not be limited and certain growth parameters can heavily fluctuate. (**b**) Alternatively, chemostat cultures can be applied, where one nutritional component is typically limited and cell density can be much higher than in shake flasks. Additionally, cell density and environmental conditions can be kept constant and more complex cultivation strategies can be implemented. (**c**) The increase of fitness during laboratory evolution experiments is fast in the first stage but generally slows down during prolonged selection, whereas the number of mutations is steadily increasing; however network complexity leads to a decreasing beneficial effect of additional mutations. [36,67]. (**d**) Mutations that are usually identified in ALE studies. Single nucleotide polymorphisms (SNPs), smaller insertions and deletions (indels) and larger deletions and insertions contribute to genetic and gene regulatory changes and fitness changes during the selection for improved phenotypes.

Generally, ALE experiments with microorganisms are easy to establish and the common methods, which are used, are shown in Figure 1a and Figure 1b. Batch cultivation in shake flasks can be performed to propagate microbial cells in parallel serial cultures. At regular intervals (usually on a daily basis), an aliquot of the culture is transferred to a new flask with fresh medium for an additional round of growth. Clearly, this easy setup has the advantages of cheap equipment and the ease of massive parallel cultures. By replacing shake flasks with deep well plates with even smaller culture volumes, hundreds of microbial cultures can be grown in parallel [44]. Several environmental factors can be easily controlled, including temperature and spatial culture homogeneity (by constant mixing of the culture). Nevertheless, batch cultivation has certain shortcomings, including varying population density, fluctuating growth rate, nutrient supply and fluctuating environmental conditions such as environmental pH and dissolved oxygen (partial oxygen pressure, pO2). In many cases, these factors may not be of great importance for the experimental setup but the simplicity of the setup can prevent the implementation of complex environments for microbial selection experiments. As an alternative to serial batch growth, continuous (chemostat) cultures in bioreactor vessels (Figure 1b) are applied [45-50]. The advantages of chemostat cultivation are constant growth rates and population densities. Furthermore, it is possible to tightly control nutrient supply and environmental conditions such as pH and oxygenation. A major drawback is the costs of operation. Even with small vessels and parallel operation, as they are available from many manufacturers, the costs are by far exceeding the costs of shake flask cultivations. A major difference between prolonged selective growth in batch and chemostat cultures is, however, the growth in nutrient-sufficient versus potentially nutrient-limited conditions. Principally, feedback control mechanisms, such as in the case of a pH-auxostat [51] allow nutrient-sufficient growth in chemostat cultures at the maximum specific growth rate; however, nutrient-limited growth at a growth rate below the maximum is predominantly applied in continuous cultures. As indicated in Figure 1a, in serial batch cultures for ALE, the transfer often occurs before the stationary phase is reached. Clearly, this is a prerequisite to avoid stationary phase adaptation [52]. Thus, microbial cells are predominantly grown in exponential cultures. In contrast, in continuous cultures, the growth rate is kept constant (or in certain experimental setups stepwise / continuously increasing) by the limitation of a major growth nutrient, such as glucose, nitrogen or phosphate. In this context, cells selected under growth limiting conditions can show growth trade-offs in non-limiting conditions and *vice versa*[53]. If the selection aims at phenotypes that do not rely on increased growth rates or biomass yield but other traits such as e.g. a yes / no survival phenotype after gene knockout or antibiotic production, solid media or combinations of solid and liquid media can be applied in order to select for proper phenotypes [54,55].

There are several critical issues that may arise during the genetic engineering and artificial selection for microbial biotechnology [16]. As example, for certain processes selection for increased tolerance to an accumulating but toxic bio-product can be appropriate. Other biotechnological processes, such as recombinant protein production and metabolite co-production in microbial cells, are coupled to microbial growth [56]. Furthermore, scale-up of microbial processes often represents a major obstacle in process design. Whereas small-scale cultivations and initial engineering steps are often performed in complex media in batch culture, during the scale-up of the process, cells are exposed to altered environmental conditions and environmental stress regimes [57,58].

Altogether, these factors should ideally also be well-considered in the case that ALE is implemented in microbial cell factory design. The initial choice of conditions, including media composition and limited versus non-limited growth rate, will greatly influence whether the artificially selected microbial host will be suitable for its dedicated application.

### Increased growth and fitness as desirable criteria

During adaptive evolution, certain trait values change and are associated with increased (Darwinian) fitness [20]; as such, an improved phenotype or property is often equal to increased fitness. During direct competition of an ancestral microbial strain and an adapted strain, the increased fitness of the adapted variant will be obvious by its increased frequency in the total population. With suitable neutral genetic markers, this fitness difference can be easily monitored during laboratory evolution of microbial cells [34,59]. Competitive fitness assays usually involve growth in batch cultures and are balanced for all growth phases (lag, exponential and stationary phase). On the other hand, the maximum growth rate (μ_max_) of microbial cells is less commonly used in order to determine changes of the evolutionary fitness. Nevertheless, for biotechnology, parameters such as μ_max_, survival rates in toxic concentrations of certain chemical compounds and absolute biomass yield are appropriate fitness criteria.

An important factor during the search for improved phenotypes by ALE is the time span for the selection experiment. In summary Tables 1 and Table 2, it can be seen that a typical ALE experiment is performed for somewhat between 100 and 2000 generations and usually takes a few weeks up to a few months. During ALE, several phenotypes will occur at first and compete for ‘dominance’ in the total population. Stable phenotypes will accumulate rapidly, although clonal interference [42], bet hedging [43,60], genetic hitchhiking [61] and fluctuating growth environments can lead to significant population heterogeneity [22,62,63]. Thus, it cannot be assumed that a homogenous population is present during any point of a laboratory evolution experiment. It should be noted that selection for improved fitness in a specialized environment often leads to significant trade-offs in other stressful or selective conditions [19]. In this context, for biotechnological purposes, the best phenotype is not necessarily the one with the highest fitness in a certain condition, but the one that shows increased performance and the least trade-offs in other environmental conditions.

**Table 1:** Adaptive laboratory evolution experiments with bacteria

**Species, Strain**	**Environment**	**Selection time**	**Reference**
**Nutrient**			
*E*. *coli* REL606	DM minimal medium, **glucose**	> 50 000 generations	Lenski et *al*. [34,36,64]
*E*. *coli* MG1655	**glycerol**-limited chemostat	217 generations	Weikert et *al*. [50]
*E*. *coli*	**glucose-**limited chemostat	280 generations	Notley-McRobb and Ferenci [49]
*E*. *coli* MG1655	M9 minimal medium, **glycerol**	700 generations	Ibarra et *al*. [65]
*E*. *coli* MG1655	minimal medium **lactate or glycerol**	>1000 and > 600 generations	Fong et al. [66]
*E*. *coli* MG1655	M9 minimal medium, **L-lactate**	900 generations	Hua et *al*. [67]
*E*. *coli* MG1655	M9 minimal medium, **lactose**	500 generations	Dekel and Alon [68]
*E*. *coli* MG1655	**glucose** minimal medium	25 days	Conrad et *al*. [69]
*E*. *coli* MG1655 ∆*pgi*	M9 minimal medium, **glucose**	50 days	Charusanti et *al*. [70]
*E*. *coli* MG1655	M9 minimal **glycerol + 1,2 propanediol**	700 generation	Lee et *al*. [71]
*E*. *coli*	chemostat minimal medium	37 days	Wang et *al*. [46]
	**phosphate limitation**		
*T*. *fusca*	Hagerdhal medium, **glucose, cellobiose**	220-284 generations	Deng and Fong [72]
*L*. *lactis* KF147	**milk**	1000 generations	Bachmann et *al*. [73]
*G*. *sulfurreducens*	**lactate**	nd	Summers et *al*. [74]
*C*. *tyrobutyricum* ATCC 25755	repeated batch fermentations, **glucose**	130 days	Jiang et *al*. [75]
*E*. *coli* REL606	DM minimal medium, **lactose**	2000 generations	Quan et *al*. [76]
*E*. *coli B*	LB **xylose, lactate production**	3 month	Zhao et *al*. [77]
**Environmental stress**			
*E*. *coli*	**high temperature** (41.5°C)	2000 generations	Rhiele et *a*l. [78]
*E*. *coli*	**UV light**	80 UV light cycles	Alcantara-Diaz et *al*. [79]
*E*.*coli*	**Freeze-thaw**	150 cycles	Sleight and Lenski [80,81]
*E*. *coli* ∆*rpoS*	**osmotic stress**	nd	Stoebel et *al*. [82]
*E*. *coli*	LB medium **heat stress 48.5°C**	620 generations	Rudolph et *al*. [83]
*E*. *coli* MG1655	rich medium , **7% (v/v) ethanol**	30 -160 generations	Goodarzi et *al*. [84]
*E*. *coli* W3110	M9 minimal medium, **5% (v/v) ethanol**	1000 generations	Horinouchi et *al*. [85]
*E*.*coli*	LB medium 4 – 8 g L^-1^**isobutanol**	45 transfers	Atsumi et *al*. [86]
*E*. *coli* EcNR1	M9 minimal medium**, 0.75% (v/v) isobutanol**	500 generations	Minty et *al*. [87]
*Bacillus boroniphilus*	TSB medium, **0.055 – 0.3 M H**_**3**_**BO**_**3**_	50 transfers	Sen et *al*. [88]
*E*.*coli* B	Davis minimal medium, **42.2°C**	2000 generations	Tenaillon et *al*. [89]
*E*. *coli* MG1655	LB medium **temperature stress (up to 48°C)**	8 months	Blaby et *al*. [90]
*E*. *coli* K-12	M9 medium, glucose,chemostat, ***n*-butanol**	> 200 generations	Reyes et *al*. [91]
*E*. *coli* MG1655	M9 minimal medium, **low pH, *n*-butanol, H**_**2**_**O**_**2**_**, high salt**	500 generations	Dragosits et *al*. [63]
**Miscellaneous**			
*S*. *clavuligerus* 27064	TSB + **MRSA N315**	nd	Charusanti et al. [55]
*G*. *oxydans* DSM2343	**glycerol,** increased DHA production	25-50 transfers	Lu et *al*. [92]

Experiments are presented in the approximate chronological order for the different selection conditions. The respective selection condition in the second column (environment) is highlighted in bold letters. nd - no data.

**Table 2:** Adaptive laboratory evolution experiments with yeasts

**Species, Strain**	**Environment**	**Selection time**	**Reference**
**Nutrient**			
*S*. *cerevisiae*	**glucose**-limited chemostat	250 generations	Ferea et *al*. 1999 [93]
*S*. *cerevisiae*	**glucose-**limited chemostat	500 generations	Dunham et al. 2002 [94]
*S*. *cerevisiae* capable of growth on xylose, EMS-mutagenized,	chemostat, minimal medium, **anaerobic growth on xylose**	170 generations	Sonderegger and Sauer 2003 [95]
*S*.*cerevisiae*	**maltose**-limited chemostat	> 25 generations	Jansen et *al*. 2004 [96]
*S*. *cerevisiae CEN*.*PK* ∆*PDC1*,*5*,*6*	chemstat, shake flask, synthetic medium**, C2-independence**	nd	van Maris et *al*. [97]
*S*.*cerevisiae*	**glucose**-limited chemostat	200 generations	Jansen et *al*. 2005 [98]
*S*. *cerevisiae* RWB 217	chemostat, batch, **xylose**	Nd	Kuyper et *al*. 2005 [99]
*S*. *cerevisiae*	synthetic medium, **arabinose**	17 transfers, ca. 3500 hours	Wisselink et *al*. 2007 [100,101]
*S*. *cerevisiae*	chemostat, sequential batch	40 days and 20 cycles	Wisselink et *al*. 2009 [102]
	**glucose, xylose and arabinose**		
*S*. *cerevisiae* DBY11331	**sulfate**-limited chemostat	188 generations	Araya et *al*. 2010 [103]
*S*. *cerevisiae* TMB3061	chemostat, synthetic medium, **growth on xylose and arabinose**	20 and 65 generations	Garcia Sanchez et *al*. [104]
*S*. *cerevisiae CEN*.*JB27* ∆*PYC1*	Synthetic medium, selection for **growth on glucose**	nd	Zelle et *al*. 2010 [105]
*S*. *cerevisiae* CEN-PK	**galactose** minimal medium	400 generations	Hong et *al*. 2011 [106]
*S*. *cerevisiae*, engineered, *scfa*^+^, Pyc^-^	batch and nitrogen limited chemostat, **anaerobic glucose**	approx. 30 days	Zelle et *al*. 2011 [107]
*S*. *cerevisiae*	**glucose** limitation	approximately 100 generations	Wenger et *al*. 2011 [53]
SC288			
*S*. *cerevisiae*	increased **xylose** fermentation	nd	Shen et *al*. 2012 [108]
*S*. *cerevisiae*, engineered	batch and chemostat cultures, **xylose utilization and ethanol production**	70 + 120 generations	Zhou et al. 2012 [109]
*S*. *cerevisiae* CMB.GS001	Increased aerobic growth on xylose	10 cycles	Scalcinati et *al*. 2012 [110]
*S*. *cerevisiae* ∆*jen1*	synthetic medium, **lactate**	10 tranfers	de Kok et *al*. 2012 [111]
*S*. *cerevisiae*	VERT, YNB, **lignocellulosic hydrolysate tolerance**	463 generations	Almario et *al*. 2013 [112]
**Environmental stress**			
*S*. *cerevisiae* CEN-PK **EMS mutagenized**	chemostat and batch selection, **multiple abiotic stresses**	up to 68 generations	Cakar et *al*. 2005 [113]
*S*. *cerevisiae* CEN-PK **EMS mutagenized**	YMM, **continuous and pulsed CoCl**_**2 **_**stress**	25 transfers	Cakar et *al*. 2009 [114]
*C*. *albicans*	**fluconazole**	330 generations	Selmecki et *al*. 2009 [115]
*S*. *cerevisiae* BY4741	YP galactose medium, **0.5 M NaCl**	300 generations	Dhar et *al*. 2011 [116]
*S*. *cerevisiae* BL7	YP medium**, 0 – 2.5 g L**^**-1 **^**CuSO**_**4**_	nd	Adamo et *al*. 2012 [117]
*S*. *cerevisiae*	SD medium, **1.17% NaCl, 37°C**	25 generations	Gray and Goddard 2012 [118]
*S*. *cerevisiae*	YP medium, **6 – 8% ethanol**	141 generations	Avrahami-Moyal et *al*. 2012 [119]
W303			
*S*. *cerevisiae*	**salt and oxidative stress**	300 generations	Dhar et *al*. 2013 [120]
**Miscellaneous**			
*S*. *cerevisiae* ∆*myo1*	**cytokinesis stress**	nd	Rancati et *al*. 2008 [54]
*S*.*cerevisiae* EC1118	SD **gluconate**,	240 generations	Cadière et *al*. 2011, 2012 [121,122]
	**enological properties**		

Experiments are presented in the approximate chronological order for the different selection conditions. The respective selection condition in the second column (environment) is highlighted in bold letters. nd - no data.

Besides the time needed to accumulate improved phenotypes, the degree of increased fitness that can be achieved, is an important factor for efficient implementation of ALE in microbial strain engineering. Based on previous studies, we can estimate that within 100 to 500 generations (corresponding to up to 2 months of selection for a typical *E*. *coli* or *S*. *cerevisiae* culture), a fitness increase of up to 50–100% can be achieved. In certain cases ALE can lead to an even higher increase of up to 1000% as recently shown for the iron reduction rate of *Geobacter sulfurreducens*[123], although this increase was only achieved after 24 months of selection (a time scale that may not be feasible for many biotechnological purposes). The maximum achievable fitness increase within a certain time or generation span can vary significantly depending on the selection pressure. Whereas certain environments can lead to fast selection of improved phenotypes, others, including the long-term adaptation to environmental pH [59,63], represent complex environments where microbial cells show low adaptive potential in terms of absolute fitness gain. In this context, we cannot clearly predict the scale at which improved phenotypes emerge during laboratory evolution, although recent literature indicates that such predictions are possible in certain growth contexts such as carbon source adaptation [65]. Moreover, it is important to note that the fitness increase as a function of the total number of generations is not linear (Figure 1c). Whereas the fitness increase is usually fast within the first 100 to 500 generations, it slows considerably down during the course of ALE [36]. For standard ALE in biotechnology this is a critical factor, as prolonged selection that exceeds the first rapid evolutionary adaptation phase will not necessarily lead to significantly improved phenotypes [67]; therefore, there is a clear cost benefit ratio of experimental selection time and achievable fitness improvements for most microbial engineering purposes. Thus, complex environments may depend on advanced selection strategies to compensate a low adaptive potential and the ceasing fitness gain during ALE.

Many typical ALE experiments were performed in shake flasks and similar growth conditions with cell densities in the range of 10^7^ to 10^9^ cells per mL for typical microbial cultures [124,125] and as such, the total number of generations is commonly used to estimate the emergence of adaptive mutations. Nevertheless, this correlation does not take into account the total number of cells in a population. Only recently, the cumulative number of cell divisions (CCD) was proposed as a more appropriate denominator to evaluate microbial evolution [126].

### Mutations and robustness

Mutations are the basis underlying genetic change and the selection of improved phenotypes in nature as well as in the laboratory. Although DNA replication itself is a process of high fidelity with a mutation rate of approximately 10^-10^ per base pair per replication for microbial cells [127], the large population sizes in microbial cultures still allow the emergence of frequent mutations within a short time span.

In recent ALE experiments various types of mutations were detected: Single-nucleotide polymorphisms (SNPs), small scale insertions and deletions (indels), transposable element (insertion sequence, IS) movements and amplifications as well as deletions of larger genomic regions (Figure 1d). Altogether, these genomic changes contribute to the change of trait values during laboratory evolution. As summarized in a recent review, SNPs (61%, with G:C to T:A mutations being disproportionally frequent) account for the majority of observed genetic changes [15]. Deletions accounted for approximately 29% of the observed genomic changes, followed by insertions (7%) and IS movements (3%) [15]. Similar to SNPs, these types of mutations also contribute to increased and decreased gene dosage, altered or diminished gene function or altered gene regulatory patterns. In several recent studies, gene amplifications were identified to contribute to increased gene dosage [63,116,128] and subsequently increased fitness. In a purely evolutionary perspective, they are even more important. Whereas there is an ongoing debate about the long-term fixation of gene duplications, either by their selective advantage under selection pressure, or by selective neutrality and fixation by random drift, they are a prerequisite for the development of novel molecular functions [129-131].

For microbial, more specifically prokaryotic cells, a new model for the emergence of novel metabolic pathways was introduced. This so-called toolbox model suggests that prokaryotic metabolic pathways emerge through horizontal gene transfer (HGT) and are rapidly acquired and lost based on nutrient availability [132]. Nevertheless, the role of HGT in natural evolution remains a controversial topic [133].

Microbial metabolic networks often provide appropriate functions at a minimal size [134] and furthermore, their complexity seems to be correlated with environmental factors [135]. If metabolic networks operate at a minimal size, the mutational robustness has to be high in order to maintain proper functions. Although counterintuitive at first, it was shown that mutational robustness can have a positive effect on evolvability, depending on the population size and network architecture [41,136]. In laboratory evolution experiments, chemical mutagens, mutants deficient in DNA repair or transposon libraries can be applied in order to speed up the selection process by increasing the diversity of targets for selection. Random mutagenesis is also a very popular mechanism in order to select for improved phenotypes in biotechnology [137,138]. Interestingly, it was shown that high mutation rates and mutator strains can emerge naturally during laboratory evolution and contribute to the formation of genetic novelty [33,73,139-141]. It is clear that a higher mutation rate can only be beneficial to a certain extent as it also results in a genetic load [142]. Recent data indicated that long-term adaptation, mediated by an increased mutation rate and genetic load, is tightly balanced in bacterial cultures. For example, in a recent *E*. *coli* study, a mutator phenotype *mutT* was invaded by *mutY* mutations. The authors estimated that the *mutT* mutation leads to an approximately 150-fold increase of the mutation rate, whereas the *mutY* mutations decreased the mutation rate by 40–60% in order to decrease the genetic load [143]. Furthermore, it was shown that mutational robustness tends to increase with mutation rate and decline with population size [40].

Considering this general tension between mutational robustness of genes and networks and the potential to evolve, enzyme promiscuity is also important for the evolution of novel functions [144]. Moreover, there is evidence that the interplay of protein mutational robustness, protein folding and environmental stress is an additional key factor that determines the evolution of new traits [145,146]. The evolution of novel functions and pathways can be hardly observed in a laboratory evolution experiment, even with microbial cells and their fast growth rates. Nevertheless, there are a few examples that highlight that a novel trait can emerge rapidly on a laboratory time scale. For example, an experimental *E*. *coli* culture could evolve the ability to aerobically utilize citrate [37], or the ability to use the non-natural carbon source 1,2-propanediol [71]; also, a strain deficient in glutathione biosynthesis was able to develop compensatory mutations to restore this function [147].

Microbial cells are generally grown at constant growth rate in chemostats or at constant μ_max_ during the exponential phase of a batch culture. It should be noted that microbial batch cultures can show rapid evolutionary adaptation if they are exposed to prolonged stationary phase. In fact, this nutrient limited condition represents a very dynamic environment for microbial populations. So-called GASP (growth advantage in stationary phase) mutants are rapidly fixed in *E*. *coli* populations [52]. Mutations in amino acid catabolic processes and stress response sigma factor, *rpoS*, are of special importance for acquiring a selective advantage in such nutrient starved conditions [148,149]; however, such mutational adaptations might be counterproductive for some applications of microbial cells in biotechnology.

## Adaptation towards nutrient sources and environmental stresses

In nature as well as in biotechnological processes, microorganisms are challenged with changing environmental conditions, where they experience fluctuations in temperature, pH, oxygenation, atmospheric or hydrostatic pressure but also water and nutrient availability; thus, the selective pressures can be divided in two main categories: nutrient availability and environmental stress [17]. Microbial cells evolved mechanisms to successfully cope with such stressful conditions in order to maintain cellular homeostasis. In contrast to natural environments, in industrial processes microbial cells may be challenged with non-natural stresses that may result in poor and non-competitive process performance. Many ALE studies analyzed the impact and molecular adaptation towards both stresses (Table 1 and Table 2), leading to major findings regarding the emergence of specialists, generalists, the underlying mutations and the applicability of evolved cells for industrial microbiology.

### Nutrient stress

Efficiency of nutrient source utilization is an important aspect of microbial growth and for industrial purposes it may be governed by factors such as substrate cost or increased bioconversion rates. Multiple studies analyzed the adaptive changes in microbial populations upon nutrient selection pressure.

In a 1997 study, *E*. *coli* was evolved in chemostat cultures in order to select for improved growth on glycerol. The authors reported phenotypic changes in terms of colony morphology accompanied with increased growth rate and biomass yield and decreased acetate formation [50]. Subsequently, it was shown that suboptimal growth on glycerol can approach its theoretical maximum during laboratory evolution [65]. In a more recent study, *rpoC* RNA polymerase (RNAP) mutations were found to be a major source for improved growth on glycerol during batch selection [69]. In this study, the biomass yield increased up to 40% and acetate overflow was reduced, whereas the metabolic rate increased and the evolved cells showed a lowered or total loss of motility. The *rpoC* mutations that were identified led to altered gene expression patterns by a global redistribution of transcriptional units (TUs) from ribosomal RNAs to other units.

Other studies focused on the adaptation of *E*. *coli* to growth on glucose. In this context the Lenski long-term experiment with 12 replicate cultures is performed with glucose as carbon source. During the first few thousand generations, glucose-evolved cultures developed larger cell size and a lower final cell density as compared with the ancestor [34]. Also, a significant parallelism in the change of the gene expression profiles was noticed in these parallel cultures [64]; WGS of the parallel populations revealed that most cultures acquired mutations in the transcriptional regulator *nadR*, the pyruvate kinase *pykF*, the *rbs* operon, the transcriptional regulator *malT* and the stringent response regulator *spoT*[36]. Furthermore, changes in DNA topology contributed to global transcriptional changes by altered DNA superhelicity, mediated by mutations found in genes such as *fis*, *topAB*, *gyrAB* and *dusB*[150]. In contrast to batch selected populations, during aerobic glucose-limited chemostat growth in parallel cultures for 280 generations, mutations in the glycoporin *lamB* and the regulator *malT* were identified as the cause of long-term adaptation. Moreover, within a single chemostat culture, the authors observed multiple stably co-existing *malT* mutations [49]. Strikingly, the authors also noticed that *malT* regulatory mutations, although common in aerobic conditions, were absent during oxygen-limited chemostat growth, where mutations in the glucose permease *ptsG* were common [48]. In another study, the adaptation of a phosphoglucose isomerase (*pgi*) deficient strain to glucose was analyzed [70]. Growth rates and glucose uptake rates increased up to 3.6 and up to 2.6 fold in evolved ∆*pgi* strains as compared with the ancestor and were accompanied with different phenotypes regarding acetate and formate production. The authors found frequent point mutations in *rpoS*, *udhA* (soluble transhydrogenase), *pntAB* and a prophage deletion. They did not observe these mutations in the glucose-adapted wildtype strain; other regulatory mutations (including *rpoABC*) occurred in both backgrounds and thereby show, how changes in the gene regulatory network lead to divergent evolutionary results. Frequent *rpoB* mutations were also found during batch evolution on glucose in a more recent study [63], but most importantly the *rpoS* mutation alone did not positively influence the growth rate in the ancestral ∆*pgi* strain; *rpoS* mutations were epistatically linked to additional mutations.

For other, tightly regulated carbon sources such as lactose in *E*. *coli*, it was shown that *lacZ* expression levels were evolutionary fine-tuned depending on the lactose concentration in the environment, in order to balance the cost of *lacZ* expression and increased fitness [68]. Moreover, recent data showed that *E*. *coli* can evolve different modes of *lacZ* expression, depending on the environment that contained either lactose, glucose or both as carbon sources. Mutations that lead to altered expression patterns accumulated predominantly in *lacI* and lacO1 regions of the *lac* operon [76]. Altogether, these mutations were responsible for a reduced (diauxic) lag phase and increased maximum growth rates in lactose growth medium.

Growth on other carbon sources such as lactate also leads to altered phenotypes, such as decreased acetate overflow in order to tune the cellular energy metabolism [67]. Phosphate limitation experiments resulted in genotypic and phenotypic divergence of *E*. *coli* through mutations in *rpoS*, *spoT* and *hfq*, which led to the de-regulation of *pho* genes and increased phosphate transport [46]. Finally, a recent study applied laboratory evolution in order to allow homofermentative L-lactic acid production from xylose in recombinant *E*. *coli*. By serial transfers the authors were able to improve anaerobic growth on xylose, although the genetic basis remains yet to be determined [77].

Similarly to *E*. *coli*, nutrient adaptation was also extensively studied in *S*. *cerevisiae*. Early studies in glucose-limited chemostats revealed that glucose-limited selection led to increased biomass yield and a decreased fermentative capacity [93,98]. Decreased activity levels of enzymes related to glycolysis but also correlating changes in the expression levels of genes such as *ENO1*, *ENO2*, *TDH1* and *PYK1* and down-regulation of the stress response (*MSN2*/*4*) were observed. Altered cell morphology was also observed, as well as higher affinity towards glucose, although no expression change in the major *HXT* low-affinity glucose transporters was observed [98]; another study reported the emergence of aneuploidy during glucose-limited growth and amplification of the *HXT6* gene with significant divergence between co-evolved populations [94]. Glucose import, sensing and regulation represent major targets during glucose-limited chemostat growth, as a further study also identified frequent mutations in *HXT* genes and glucose sensors and regulators such as *RGT1* and *MIG2*[53]. In another study, van Maris and co-workers evolved a *S*. *cerevisiae* strain with deficient pyruvate decarboxylase activity for high-glucose tolerance [97]. Subsequently, it was discovered that this glucose tolerant phenotype was due to an in-frame deletion in the glucose regulator *MTH1*, which led to altered stability of the *MTH1* encoded protein [151]. Similarly, Zelle and co-workers established growth on glucose by genetic- and evolutionary engineering in a pyruvate carboxylase-negative *S*. *cerevisiae* strain. The authors identified a point mutation in the recombinant *sfcA* gene, which shifted the co-factor preference from NADH to NADPH [107].

For other carbon sources, it was shown that selection for improved galactose utilization could be achieved by serial transfer with a 24% increase of μ_max_ within 62 days. Galactose uptake rates and ethanol production were increased. Transcript analysis showed up-regulation of trehalose and glycogen metabolism, whereas no mutations were identified in galactose or storage carbohydrate related genes. Interestingly, the authors rather identified mutations in RAS/PKA signaling as a source for increased fitness on galactose medium [106].

In xylose adaptation studies, increased growth rates and higher ethanol yields were observed in recombinant yeast. In this case, trehalose and glycogen metabolic processes were significantly down-regulated, nutrient and stress signaling was affected by down-regulation of *YAK1* and also accompanied by down-regulation of *CWP1* and consequently major cell wall changes [108]. The authors did not identify expression changes in *HXT* genes, although other studies on improved xylose and arabinose utilization indicated that *HXT* genes may also be involved in increased xylose uptake rates [99,104]. Furthermore, experiments on pentose utilization highlighted that increased gene copy numbers of the heterologous genes in recombinant *S*. *cerevisiae* can emerge during evolutionary engineering and contribute to increased growth in anaerobic conditions [104,109]. Finally, another example of nutrient stress selection in *S*. *cerevisiae* showed that sulfate limitation led to mutations affecting TOR signaling via an *RRN3* mutation and a genomic amplification of the high-affinity sulfate transporter *SUL1*[103].

Although rare at this point, at least one evolution experiment showed that the general principles that were observed for *E*. *coli* and *S*. *cerevisiae*, including the tendency towards optimized biomass yield and overflow metabolism, large-scale regulatory changes and mutator strains also emerge in other microbial hosts, such as *Lactococcus lactis*[73].

### Environmental stress

Among environmental stresses, adaptation to temperature has been investigated in multiple *E*. *coli* studies. Riehle and co-workers reported that heat-inducible genes, including *hslT*, *fkpA* and *gapA*, showed adaptive expression changes during prolonged exposure to increased temperature [78]. In another study it was found that the adaptation to extreme temperature relied on the constitutive expression of *GroEL*/*ES* in *E*. *coli*[83], whereas a similar study with a different selection procedure identified mutations in *glpF* and *fabA*, accompanied with increased membrane fatty acid saturation as critical factors towards increased thermo-tolerance [90].

Increased growth during osmotic stress was linked to mutations that led to increased expression of enterobactin biosynthesis genes (*fepA* and *entD* among others) [63]. Furthermore, it is known that *rpoS* plays a crucial role towards survival in high osmotic conditions. It was shown that an *E*. *coli* population with a *rpoS* deletion can still develop increased salt tolerance during laboratory evolution by uncoupling of *rpoS* dependent expression of the *otsBA* operon, which is important for trehalose synthesis [82].

Apart from temperature and salt stress adaptation, organic solvent tolerance has been a major research topic in recent years, due to its importance for next generation biofuel production. Adaptive changes towards ethanol tolerance were linked to the up-regulation of *oxyR* and *nrdR*, indicating major changes in the cellular respiratory system but also changes in amino acid metabolism [85]. Furthermore, changes in enterobactin biosynthetic processes (*ent* genes) were also observed in independent studies [84,85,91]. Increased ethanol tolerance in *E*. *coli* can also be mediated by increased catabolism of ethanol through the TCA cycle [84]. Three other studies dealt with the adaptive evolution towards increased iso- and *n*-butanol stress [63,86,87]. Mutations in *acrAB* and *marC* were found independently in these studies; further changes were linked to carbon and nitrogen metabolism via *gatY* and *tnaA*[86], enterobactin synthesis [63] or attenuated *rpoS* activity via *hfq* mutations [87]. Moreover, mutations identified in isobutanol-resistant strains showed a high degree of epistasis [87]. The occurrence of enterobactin related mutations in multiple ALE studies highlight the importance of tuning the cellular redox machinery during environmental stress exposure of *E*.*coli*. Furthermore, increased oxidative stress resistance in *E*. *coli* may be linked to mutations in the *soxR* reducing system and increased levels of cellular catalase/peroxidase (*katG*) [63].

In the model yeast *S*. *cerevisiae*, adaptation towards salt tolerance led to increased cell size and increased ploidy. Additionally, gene expression changes in *CTT1* and *MSN4* were observed, as well as a high-frequency SNP in the transcriptional regulator *MOT2*[116]. In another study, increased ethanol tolerance was mediated by mutations in the translational regulator *SSD1* and *UTH1*, a protein of unknown function, and indicated that cell wall stability is a major factor in ethanol tolerance of *S*. *cerevisiae*[119]. Increased copper tolerance was reported to be mediated by a genomic amplification of *CUP1*, decreased basal levels of the copper transporters *CTR2* and *CCC2* and decreased activity of antioxidant enzymes [117,128]. Increased tolerance of *S*. *cerevisiae* towards lignocellulosic hydrolysates such as acetic acid and furfural was correlated with adaptive changes of the oxidative stress response [112].

Altogether, large scale transcriptional re-arrangements and gene amplifications are common during laboratory evolution. As such, ALE studies highlight that duplications and large scale genomic variations, although they can lead to significant issues such as genomic instability [152], play a major role in e.g. yeast evolution; this is also indicated by data on wine yeasts, galactose utilization, comparative genomics and drug resistance [115,153-156]. From these data it can be concluded that: (a) long-term adaptation leads to both large-scale transcriptional de-regulation and optimization of terminal nodes (fine-tuning of the expression levels of individual genes), (b) although parallelism is often observed in replicate cultures or even among different laboratories, different evolutionary paths (divergence) can lead phenotypic convergence and (c) epistasis is a major factor for adaptive optimization of gene regulation [89,157]. Importantly, epistatic interactions can be hardly inferred from metabolic modeling studies although significant epistatic connections for strain engineering may be easily identified in ALE studies.

### Evolutionary trade-offs and cross-protection

Microbial cells have an intrinsic capacity to balance self preservation (stress protection) and nutritional competence (SPANC). This SPANC balance was intensively studied in *E*. *coli* and is governed by strict control of processes such as stress and stringent response [158,159]. Recently it was also shown that yeast has a similar intrinsic sensing mechanism to balance growth and stress resistance [160,161]. Evolutionary studies as described above highlight how transcriptional changes can shift the balance from one to the other in order to shut down costly but gratuitous processes. In this context, transcriptional changes during laboratory evolution and specialization can lead to trade-offs or cross-benefits in alternative environments.

Glycerol-limited growth led to stable mutants in a single *E. coli* population, where one mutant showed increased growth in glycerol-sufficient batch cultures and a second mutant did not. Furthermore, the mutant that showed increased growth in batch culture also showed increased fitness under general nutrient limitation, heat and osmotic stress [50]. On the other hand, long term batch cultivation on glucose led to a rapid decay of non-used metabolic functions, including growth on D-ribose and L-glutamine, in *E*. *coli*[35], whereas other studies reported improved growth on many non-evolutionary carbon sources after batch selection with gluconeogenic carbon sources [66,67].

Similarly, aerobic chemostat-selected *S*. *cerevisiae* cells also showed increased fitness in anaerobic glucose-limited chemostats and in acetate-limited chemostats, but decreased fitness in nitrogen-limited batch cultivations [53]. Yeast cells that were evolved for efficient galactose utilization [106] subsequently showed trade-offs when grown on glucose as carbon source. This effect was due to antagonistic pleiotropy that was based on mutations of the regulatory protein *RAS2*[162].

Regarding the adaptation towards environmental stresses, it was shown for *E*. *coli* that increased ethanol tolerance comes at the cost of decreased resistance to acidic conditions [84] and *n*-butanol tolerance is weakly compatible with oxidative stress [63] and leads to trade-offs in hexane and chloramphenicol resistance [86]. Furthermore, strains evolved under phosphate limitation showed trade-offs in sodium chloride and oxidative stress survival due to decreased *rpoS* levels [46].

In contrast, it was shown for low temperature evolved *E*. *coli* populations that trade-offs at non-evolutionary high temperatures are not universal as individual lineages showed no trade-offs or even a fitness increase [163]. Similarly, *n*-butanol tolerance was reported to be highly compatible with osmotic stress [63].

Examples for adaptive cross-protection are also available for *S*. *cerevisiae* where cobalt resistance also improved tolerance for other metal ions and pulsed cobalt stress exposure during laboratory evolution even resulted in evolutionary cross-protection to thermal and oxidative stress [114]. A recent study showed that cross-protection towards lignocellulosic hydrolysates can be evolved in *S*. *cerevisiae*. Interestingly, several mutants showed increased fitness in the presence of inhibitor combinations but reduced fitness when exposed to individual hydrolysate components [112]. Another interesting study analyzed to which extent multi-stress resistant *S*. *cerevisiae* populations can be evolved. The authors tested various chemostat and batch stress regimes and found that batch selection for freeze-thaw tolerance also selected for increased thermal, oxidative and ethanol stress resistance [113].

Thus, evolutionary trade-offs and cross-protection against process-relevant parameters are ubiquitous, but the mode of cultivation and the specificities of the selection pressure largely determine to which extent these phenomena arise.

## Non-conventional hosts – implications of ecological niches and genetic network architecture

Mesophilic organisms such as *E*. *coli* and *S*. *cerevisiae* have inherent properties that limit their use in certain applications, e.g. high temperature processes. Therefore, non-conventional microbial species play an important role in biotechnology [164-168].

**Figure 2:**
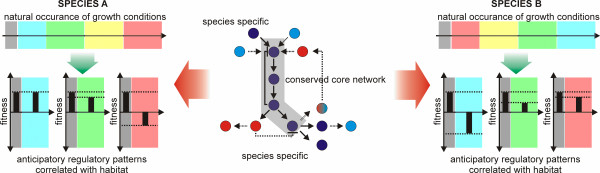
**Species-specific differences in gene regulatory networks.** Species-specific network properties allow for anticipatory behavior of the environment and consequently species-specific fitness trade-offs, in case that environmental stresses do not occur as in their natural order. Ultimately, these network properties may lead to distinct trade-offs among non-conventional host organisms during laboratory evolution.

Currently, limited data on the physiology of such organisms still represent a significant obstacle towards rational host cell engineering. Although there are many similarities in the biochemical networks across most microbial hosts, it is well documented that species and environmental niche-specific differences evolved. Whereas core sub-networks and pathways tend to be well conserved, the up- and downstream connections to other cellular networks vary significantly among different species and lead to species-specific differences of stress resistance in adverse environments [169,170] (Figure 2). The evolutionary mechanisms described in the preceding sections are crucial towards the development of such specializations [130,156,171]. They allow anticipatory and foraging behavior in pro- and eukaryotic microbial cells [172-174]. In fact, a recent study in *S*. *cerevisiae* indicated that anticipatory gene regulatory patterns can evolve under cycling salt and oxidative stress conditions on a laboratory time scale within 300 generations and that cross-stress protection against salt and oxidative stress is strikingly asymmetrical, with oxidative stress protecting against salt stress but not *vice versa*[120]. Another recent study in *E*. *coli* showed that, by using increased genotypic diversity leveraged by a transposon library, evolutionarily ‘old’ anticipatory responses can be rapidly decoupled [172].

Certain functions such as overflow metabolism in *S*. *cerevisiae* may have evolved in order to provide an evolutionary advantage in specific environments and to protect carbon resources from other competing species [175]. Consequently, there are differences in the regulatory blueprints of different species [169,176,177], which can ultimately lead to distinct evolutionary stress trade-offs during selection. Towards this end, a recent study with *S*. *cerevisiae* and *Saccharomyces paradoxus* showed that the evolutionary rescue (ER) frequency was positively correlated with stress concentration during 100 generations of evolution in *S*. *cerevisiae* but negatively correlated in *S*. *paradoxus*[44].

It can be concluded that insights into the adaptive cross-stress dependencies of one organism cannot infer the dependencies in a second organism with a distinct evolutionary background. As such, biotechnologists have to be aware that different (evolved) microbial hosts are likely to show distinct trade-offs during process-related stress exposure.

## Future directions

**Figure 3:**
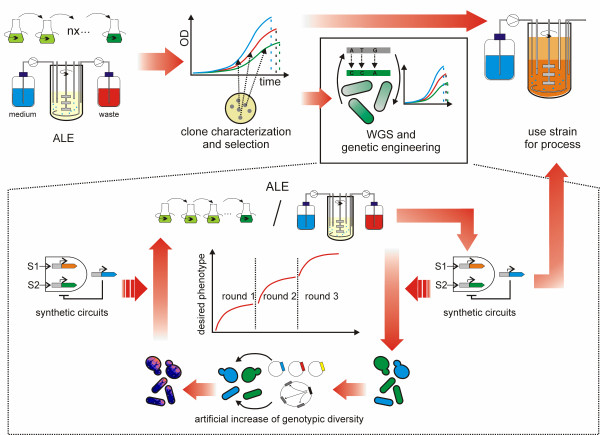
**Adaptive laboratory evolution for microbial biotechnology.** After laboratory evolution, clone analyses and selection, a suitable clone can be directly used for the desired process. Alternatively, the identification of the genetic basis of the improved phenotype can be combined with genetic engineering. The fitness increase tends to slow down during ALE, due to inherent properties of biological networks and molecular evolution. In order to allow for efficient strain engineering, laboratory evolution may be combined with classical genetic engineering tools (e.g. transposon libraries, over-expression libraries and genome shuffling). Short sequential rounds of artificial selection and *in vitro* genetic manipulation can be applied in order to obtain the desired phenotype more efficiently. Novel genetic circuits and synthetic elements for product formation and complex microbial behavior can be introduced into the ancestral or evolved cell factory.

Artificially-evolved microbial cells can be directly used for biotechnological processes (Figure 3). However, based on the data that are available to date, the direct usage comes with certain drawbacks. Biological engineers aim at improving cellular phenotypes by targeted genetic engineering and an evolved microbial strain can impair such engineering approaches because of its unknown genotype, which bears the risk of e.g. hitchhiking of unfavorable mutations that can have adverse effects during environmental stress exposure. In many respects, the same aim can be achieved by ALE and classical genetic engineering. For example, laboratory selection can lead to large-scale rewiring of gene regulatory networks, but an approach, that the authors called gTME, global transcription machinery engineering, can also yield similar results [2]. Nevertheless, with laboratory evolution balanced protein expression levels may be achieved more easily than with standard molecular biology techniques. Moreover, for eukaryotic host organisms such as yeasts, alternating asexual and sexual propagation during selection can be applied in order to decouple beneficial and unsound mutations and thus reduce potential deficits of laboratory evolution experiments [118]. Nevertheless, efficient evolutionary engineering of industrial *S*. *cerevisiae* strains has been suggested to be limited by polyploidy, due to increased genetic robustness as compared with haploid laboratory strains [104,178].

In this context, laboratory evolution can be combined with other methods in order to achieve effective biotechnological engineering (Figure 3). Some recent studies highlight the suitability of a highly integrated approach. For example, several studies improved the carbon source utilization of *S*. *cerevisiae* strains by genetic engineering in combination with evolutionary adaptation [104,107-109]. Trait selection during laboratory evolution can be restricted by the limited number of beneficial mutations that occur on a reasonable time scale. *In silico* experiments indicate that stepwise evolution can increase microbial evolvability [179]; as such, sequential short rounds of evolution may be used to increase the efficiency of ALE. The genotypic diversity can also be increased to speed up evolution. In a recent study towards increased *n*-butanol tolerance in *E*. *coli*, a drastic increase in solvent tolerance was rapidly achieved by combining laboratory evolution and genome shuffling of the evolved clones [91]. Other studies used ethylmethanesulfonate (EMS) treated *Bacillus boroniphilus* to select for increased boron resistance [88] or EMS-treated *S*. *cerevisiae* in order to achieve increased nutrient utilization and stress resistance [95,113,114]. Similarly, MAGE (multiplexed automated genome engineering) technology makes use of massively increased genotypic diversity in order to generate improved phenotypes such as improved lycopene production in *E*. *coli* and accelerate evolution [180].

Microbial gene regulatory networks are generally very robust towards perturbations and large-scale re-wiring results in a significant number of viable clones with increased evolvability as compared with the wildtype [181]. In this context, a heterologous transcriptional regulator from *Deinococcus radiodurans* was recently introduced into *E*. *coli* in order to select for improved phenotypes [182] and such extensive perturbations of regulatory networks may greatly expand our possibilities in rapid laboratory evolution towards complex traits. Another prospective approach is the co-cultivation of multiple organisms. Besides insights into co-evolutionary behavior, this approach can be exploited in order to increase antibiotic production such as in the case of co-cultivation of *Streptomyces clavuligerus* and *Staphylococcus aureus*[55]. Furthermore, in the last decade synthetic biology emerged as a very promising route in biological engineering. In contrast to classical approaches it is highly structured and biological networks are split into smaller functional modules [11,183], leading to complex optimization needs. Nevertheless, Darwinian selection and adaptive evolution has also been proposed as a potential optimization strategy in this field [184].

Finally, the implications of computational biology for laboratory evolution have to be addressed. Full-scale models of microorganisms are available [185,186] and we can simulate microbial growth. Consequently, genome scale pathway modeling itself proved extremely powerful for metabolic engineering of microbial cells [187]. Similarly, *in silico* approaches are widely applied in order to address evolutionary questions [40,172]. Computational models are well-suited to study evolutionary change on the population level, whereas *in silico* evolution of artificial cells that include simulated regulatory networks are currently very limited [21]. Enormous computational power is necessary to simulate living cells, even on a non-evolutionary scale. Additionally, even for well-studied organisms such as *E*. *coli* and *S*. *cerevisiae*, there is still a significant number (approximately 30%) of genes with unknown function. Furthermore, the complex interactions of living organisms with their environment are not well understood. Specifically, our understanding of the effect of many environmental stressors on cells, their precise action on certain cellular components, the mode of stress sensing, cellular signal transduction and cross-talk between signal transduction pathways is still limited. These deficits impede the correct implementation of such constraints into computational models. In this context, computational approaches provide important benefits towards rational engineering of microbial cell factories but as it can be foreseen right now, they cannot replace or prevent ‘trial and error’ laboratory experiments for the foreseeable future.

## Conclusions

As François Jacob pointed out: “Evolution is a tinkerer”, and as such evolution is constrained to work on available blueprints. However, genetic engineers have the possibility to rapidly construct novel circuits. The integration of adaptive laboratory evolution into metabolic engineering of microbial cells offers tuning possibilities at multiple levels of the engineering process. Towards this end, future biological engineering studies will also greatly benefit from the insights into microbial physiology that were already acquired by adaptive evolution experiments. Nevertheless, it seems necessary to expand such studies to non-conventional organisms. By combining two worlds, where we rely on the innovative power of the human mind and nature’s inherent ability to optimize existing building blocks in a non-directional manner under selective pressure, significant advances in microbial cell factory design can be expected.

## Abbreviations

ALE: Adaptive laboratory evolution; CCD: Cumulative number of cell divisions; DHA: Dihydroxyacetone; EMS: Ethylmethanesulfonate; ER: Evolutionary rescue; GASP: Growth advantage in stationary phase; GSR: General stress response; gTME: Global transcription machinery engineering; HGT: Horizontal gene transfer; indel: Insertion / deletion; IS: Insertion sequence; MAGE: Multiplex automated genome engineering; NGS: Next generation sequencing; RNAP: RNA polymerase; rRNA: Ribosomal RNA; SNP: Single nucleotide polymorphism; SPANC: Self preservation and nutritional competence; TU: Transcriptional unit; VERT: Visualizing Evolution in real-time; WGS: Whole genome sequencing.

## Competing interests

The authors declare that they have no competing interests.

## Authors’ contributions

MD and DM wrote the manuscript. Both authors read and approved the final manuscript.
